# Dynamic Reconfiguration of Security Policies in Wireless Sensor Networks

**DOI:** 10.3390/s150305251

**Published:** 2015-03-04

**Authors:** Mónica Pinto, Nadia Gámez, Lidia Fuentes, Mercedes Amor, José Miguel Horcas, Inmaculada Ayala

**Affiliations:** Departamento de Lenguajes y Ciencias de la Computación, University of Málaga, Andalucía Tech, Málaga 29071, Spain; E-Mails: nadia@lcc.uma.es (N.G.); lff@lcc.uma.es (L.F.); pinilla@lcc.uma.es (M.A.); horcas@lcc.uma.es (J.M.H.); ayala@lcc.uma.es (I.A.)

**Keywords:** FamiWare, INTER-TRUST, self-protection, self-adaptation, security frameworks, dynamic software product lines

## Abstract

Providing security and privacy to wireless sensor nodes (WSNs) is very challenging, due to the heterogeneity of sensor nodes and their limited capabilities in terms of energy, processing power and memory. The applications for these systems run in a myriad of sensors with different low-level programming abstractions, limited capabilities and different routing protocols. This means that applications for WSNs need mechanisms for self-adaptation and for self-protection based on the dynamic adaptation of the algorithms used to provide security. Dynamic software product lines (DSPLs) allow managing both variability and dynamic software adaptation, so they can be considered a key technology in successfully developing self-protected WSN applications. In this paper, we propose a self-protection solution for WSNs based on the combination of the INTER-TRUST security framework (a solution for the dynamic negotiation and deployment of security policies) and the FamiWare middleware (a DSPL approach to automatically configure and reconfigure instances of a middleware for WSNs). We evaluate our approach using a case study from the intelligent transportation system domain.

## 1. Introduction

Recent advances in wireless communication technologies and applications have enabled the large-scale deployment of wireless sensor networks (WSNs). These networks have applications in many important and critical areas, such as military, critical infrastructure monitoring, traffic control, healthcare, environment monitoring and manufacturing [1]. Security and privacy are critical issues for most of these sensor network applications. However, providing security and privacy to WSNs is very challenging due to the heterogeneity of sensor nodes and their limited capabilities in terms of energy, processing power and memory. Thus, generic security solutions do not fit well with the special requirements of WSNs. This problem can be tackled from two different points of view.

On the one hand, there is currently an important effort being made to develop specific algorithms for WSNs in the fields of trust, security and privacy [1], such as new algorithms for authentication [2], positioning [3], network intrusion detection [4], efficient key management for dynamic WSNs [5], *etc*. Since these algorithms need to run in a myriad of sensors with different low-level programming abstractions, limited capabilities, different routing protocols, *etc.*, new algorithms are continuously appearing that improve upon previous ones. This means that sensor network applications are currently in need of being self-adapting to these changes with the lowest impact for users and without stopping the devices. Additionally, these applications need to react to changes in the environment by changing the security policies that are currently deployed in the running applications. Ideally, such applications should be capable of self-protection in the sense that they could dynamically self-adapt the algorithms used to provide security.

On the other hand, we need to consider the security of WSNs also from a software engineering point of view. Software development processes should provide security solutions that take into account the high heterogeneity and variability of sensors devices and applications and also the high variability of both security requirements and solutions. The use of advanced software engineering technologies could hide the complexity of WSNs from software developers and could also deal with the automatic adaptation of the applications when the security and privacy requirements change. Dynamic software product lines (DSPLs) [6,7] allow managing both variability and dynamic software adaptation, so they can be considered a key technology in successfully developing self-protected WSNs' applications. In this paper, we propose a self-protection solution for WSNs based on the use of DSPLs. Basically, DSPLs produce software products capable of adapting to requirements that may change at runtime, by modeling the elements that could be adapted dynamically as dynamic variation points. The runtime adaptation of security policies for heterogeneous systems implies both the modeling and the dynamic negotiation of security policies. According to the literature on security solutions for pervasive applications, there is a lack of powerful techniques to address the problem of security policy negotiation, interoperability and dynamic enforcement.

In this paper, we address all these issues by combining the INTER-TRUST (INTERoperable TRUST Assurance Infrastructure)security framework [8] and the FamiWare middleware [9]. INTER-TRUST is a dynamic and scalable framework to support trustworthy services and applications for heterogeneous networks and devices. It is mainly based on the dynamic enforcement of interoperable and changing security policies [8] by modeling secure interoperability policies with different constraints. As far as we know, INTER-TRUST is the only existing framework for the dynamic negotiation of security policies in heterogeneous environments. However, INTER-TRUST does not deal with the structural variability of WSNs, where there are many sensors of different types (such as the MICAz, a 2.4 GHz, IEEE 802.15.4 compliant mote module used for enabling low-power, wireless, sensor networks, or the Java SunSPOT, the Sun Small Programmable Object Technology) and each sensor can have its own configuration. This is one of the main contributions of FamiWare. FamiWare is a reconfigurable family of middlewares for ambient intelligence (AmI) systems, including WSNs. Using a DSPL approach, FamiWare is able to automatically configure and reconfigure instances of the middleware for all of the nodes of a WSN. Thus, FamiWare solves the problems of modeling and reasoning about WSN heterogeneity and variability. In our approach, we combine both approaches to enable the generation of security solutions for heterogeneous sensor networks, where: (1) the security can be negotiated at runtime (capability provided by INTER-TRUST); (2) the security variability is formally modeled (capability provided by FamiWare); (3) the system is reconfigured to enforce the new security policies dynamically at runtime (capability provided by FamiWare and INTER-TRUST); and (4) the system reconfiguration is customized for each heterogeneous device in the AmI system (capability provided by FamiWare).

We will show how the INTER-TRUST framework can be instantiated into an instance of the FamiWare middleware generated for WSNs using a contextual advisory speed application for intelligent transport systems (CAS/ITS). The rest of the paper is organized as follows. After this Introduction, Section 2 introduces the background information required to understand our approach and the case study that we are going to use. Then, Section 3 presents the main challenges that need to be solved so as to provide security in WSNs and briefly describes our approach to address these challenges. Sections 4 and 5 present the details of our approach, including the steps required to integrate INTER-TRUST and FamiWare and the dynamic reconfiguration process in FamiWare. The evaluation of our approach is discussed in Section 6. Finally, Section 7 compares our approach with related work, and Section 8 presents our conclusions and future work.

## 2. Background

This section introduces the background required to understand the approach presented in this paper. It is basically about dynamic software product lines and dynamic security frameworks. We focus on how these techniques are used in FamiWare and INTER-TRUST, respectively. Additionally, we present the case study used throughout the paper.

### 2.1. Dynamic Software Product Lines in FamiWare

**Figure 1.**
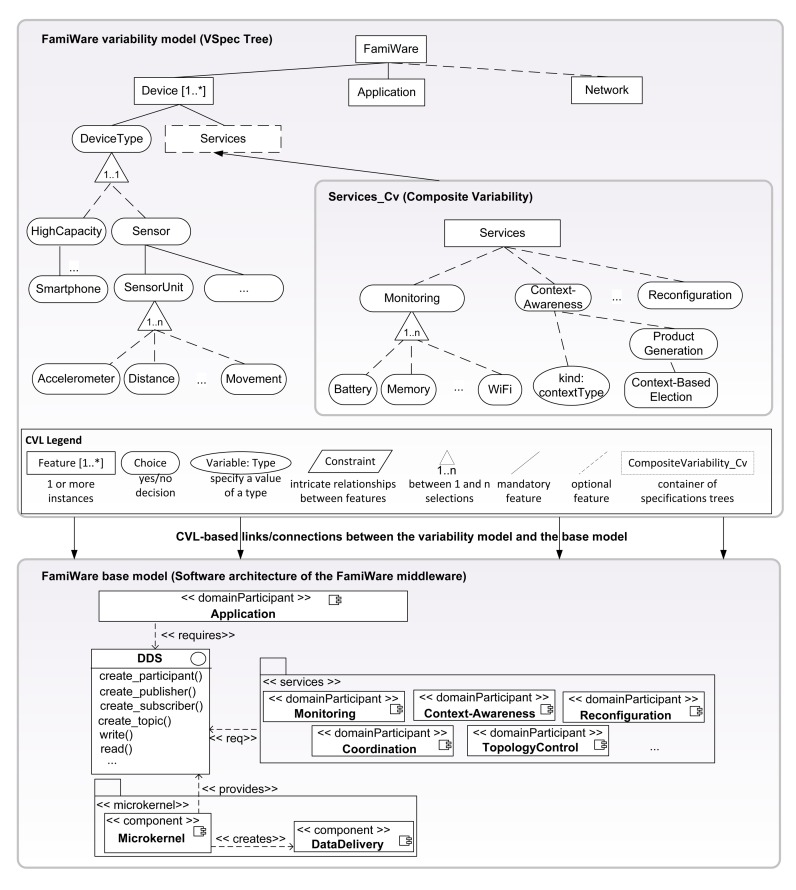
FamiWare variability model.

Dynamic software product lines (DSPLs) [6,7] represent an emerging field that is producing software products capable of adapting to requirements that change at runtime. A product is defined as a unique composition of features from a set of common and variable features (*i.e.*, variants), where some of the variable features may vary at runtime (*i.e.*, variation points). Therefore, the products have the capacity to reconfigure themselves by binding variation points at runtime, several times, in accordance with the changes in the environment. DSPLs use a variability model to specify the variation points that can be dynamically modified. In particular, in our approach, the use of non-standard languages or formalisms is avoided, and we use the Common Variability Language (CVL) [10] proposed as the standard by the Object Management Group (OMG) [11]. CVL is a domain-independent language for specifying and resolving variability. The CVL variability models allow modeling the variability separately from the base model, but both the variability and the base models are connected and can be managed using the same tool (see Figure 1). In CVL, VSpecs (Variation Specifications) are tree structures representing choices or elements (called “features” in most SPL terminologies) and can include logical constraints defined in a subset of the Object Constraint Language (OCL) [12]. The main characteristics of CVL that we use to model the FamiWare variability model are shown in Figure 1. In DSPLs, a variability model configuration is the set of features currently bound at runtime that represents a particular product of the SPL. In this way, dynamic reconfiguration is defined in terms of replacing the current variability model configuration with a new configuration in which the variation points have been re-bound to adapt them to a context change. Therefore, using the CVL approach, we automatically generate both the initial configuration and also the successive adapted configurations.

As shown in Figure 1, the inherent heterogeneity and the variability of WSNs are modeled in FamiWare using CVL variability models (*i.e.*, the VSpec trees). FamiWare proposes the customization of the piece of software related to the middleware platform that is deployed in each device of the WSN according to: (1) the device features (device [1..*] feature in Figure 1 with a 1..* cardinality to represent the different devices in the system); (2) the application requirements (application feature); and (3) the global characteristics of the network used to connect the different devices (network feature). Each device is represented, among other features, by its type (choice DeviceType) and the FamiWare services that need to be instantiated in that particular device according to the application's requirements and the network's characteristics (service choice). Regarding their types, devices can be of high capacity (such as desktop computers or smartphones) or they can be sensors (sensor choice). The sensors, among other things, have one or more sensor units (choice SensorUnit) (e.g., accelerometer, distance or movement sensors). For FamiWare, the variability is decided for each device, so the FamiWare services instantiated in each device can be different. Among other services, FamiWare is composed of three optional services: monitoring, context awareness and reconfiguration. The FamiWare monitoring service observes different types of contexts (e.g., memory consumption, battery level, status of network connection). These contexts are optional choices that will be selected or not in a particular configuration depending on the capacities of the sensor nodes. The context awareness service receives events with the monitored data (modeled as CVL variables in Figure 1) and checks whether these data imply that a context change has occurred (e.g., the memory consumption of a sensor is very high). When a context change is detected, the reconfiguration of FamiWare may be necessary to maintain proper functioning (e.g., modifying the tasks of certain sensors to reduce memory consumption). In order to do that, the context awareness service, by means of its product generation module, triggers the automatic election (context-based election) of the new variability model configuration adapted to the new context. This new middleware configuration is automatically generated by FamiWare (e.g., a configuration with a different security algorithm that consumes less). This is done at the model level giving as input the new context conditions and using the dependencies between features or the cross-tree constraints. The configuration that deals with these context conditions is automatically calculated by CVL. Then, a reconfiguration plan with the implementation level tasks for switching from the previous configuration to the new one is also generated by FamiWare. For that, FamiWare takes as inputs the previous running configuration and the next one, uses a difference operator (which has been previously implemented) to obtain the differences between the two configurations and generates the plan. Finally, this plan is interpreted and executed in the sensors by the reconfiguration service. Note that Figure 1 shows a reduced version of the complete FamiWare variability model, where the details of the application and the network features, among other things, have been omitted.

The FamiWare base model consists of a microkernel plus services architecture, as shown in the lower part of Figure 1. The composition between services and the application and between the services themselves is performed with a publish/subscribe event-based mechanism, specifically a reduced implementation of the data distribution service (DDS) [13], which is an OMG standard for publish/subscribe middlewares.

### 2.2. Dynamic Security Frameworks: INTER-TRUST

A security framework is a reusable set of modules that can be customized to develop trustworthy applications. The framework describes the interfaces and main functionality of each module, as well as the flow of control between them [14]. There are two different types of modules that can be distinguished in a security framework. On the one hand, the framework contains a set of modules that implement the security concerns, such as authentication, authorization, privacy, encryption, *etc*. On the other hand, the framework contains modules to deploy and manage the security of an application (*i.e.*, when and how the security concerns are incorporated into an application). From among these modules, the framework can incorporate specific modules to adapt the security level of the applications at runtime. In this case, we are referring to dynamic security frameworks.

**Figure 2.**
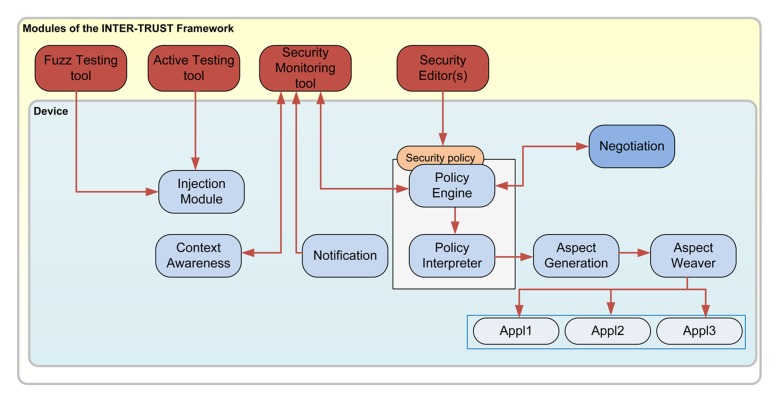
The INTER-TRUST framework.

The INTER-TRUST framework is a dynamic security framework to support trustworthy applications based on the enforcement and dynamic adaptation of security policies. As shown in Figure 2, in the INTER-TRUST framework, security policies are first specified using a security editor (e.g., MotOrBac, an Organization-Based Access Control policy editor [15]) and then negotiated between the different parties in a communication using a negotiation module. When the security requirements change at runtime, the security policies are (re)negotiated. The negotiated security policy is then analyzed and interpreted by the policy engine and the policy interpreter modules. These modules are responsible for identifying changes in the security policy that require adapting the security concerns (e.g., authentication, encryption, *etc.*) deployed inside the application dynamically, at runtime. When security policies need to be dynamically deployed and/or reconfigured at runtime, the aspect generation and the aspect weaver modules are in charge of obtaining the differences between the old and the new security configurations. Additionally, these modules incorporate or eliminate the corresponding security modules in the application itself. The environment is monitored using the notification module and the Security Monitoring tool. The monitored information is used by the context awareness module to notify the changes in the context. These changes can generate a new negotiation of the security policy and the negotiation-interpretation-deployment process begins again. Changing the security at runtime can introduce security flaws into the application. In order to avoid this, different active and fuzz testing techniques (Fuzz Testing and Active Testing tools) are provided as part of the framework in order to confirm that the dynamic changes are not harmful.

### 2.3. Case Study: Intelligent Transportation Systems

One of the most relevant applications today, where the security issues in WSNs play a very important role, are the intelligent transportation systems (ITS) [16]. These systems have the potential to improve mobility, safety and security, while ensuring energy efficiency and reducing the environmental impact of transportation systems. These systems are composed of different kinds of devices installed at several locations, such as inside the vehicles, on the road or in the traffic signals. For instance, the vehicles can be equipped with several sensors with different sensor units, such as acoustic, accelerometers, temperature, the vehicles' on-board unit (OBU) computers and GPS devices. These sensors are used to ensure safe driving, being responsible, for instance, for measuring the distance to obstacles around them. The vehicles also use information received from surrounding vehicles, such as their speed or distance, and are able to communicate with each other in order to cooperate for safety and other reasons. Furthermore, the traffic signals send information to the vehicles informing them of speed limits and general traffic regulations. ITSs have a wide variety of interesting applications, such as road safety applications (accident warnings, red-light warnings, road congestion, contextual speed advisory, *etc.*), information dissemination applications (parking spots, traffic warnings/conditions, fuel prices, landmarks, *etc.*), entertainment applications (file sharing, advertisements, voice communication with vehicles nearby, *etc.*) or assisted driving. Additionally, vehicles can be considered as distributed sensors that can collect environmental data (such as average speed, potholes, temperature, pollution, *etc.*) and send a report to base stations. This infrastructure composed of the vehicles' sensors, GPS and OBUs, road sensor units (RSUs) and other sensors or actuators installed in traffic signals, is common to all of these applications. Similarly, some of the requirements, such as coordination, monitoring or security, are also common to all of the applications.

In particular, the security needs of all of these applications are quite important, especially when dealing with safety-related services. In these cases, to know whether a node in the network is being uncooperative or malicious is really crucial to prevent intentionally wrong instructions being sent to a vehicle. Furthermore, these systems are very dynamic, and their contextual environment changes very frequently, so they need different security policies adapted to the changing situations. Additionally, the ITS infrastructure is composed of extremely heterogeneous devices where both the monitoring and the deployment and management of the security policies must be performed in different ways.

**Figure 3.**
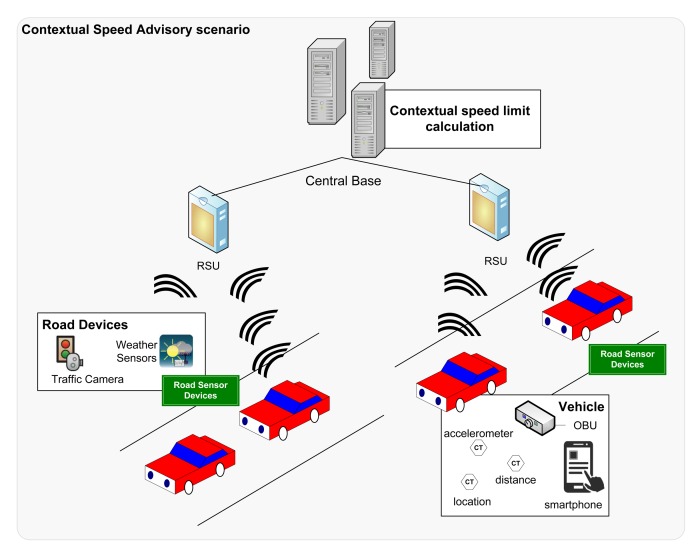
A context speed advisory (CSA) scenario.

In this paper, we illustrate these needs with a specific ITS application, a contextual speed advisor (CSA) application [16], where vehicles are informed about the speed they have to follow, taking into account the contextual environment. As shown in the scenario in Figure 3, this kind of application has the same common infrastructure as previously described, with sensors, OBUs, base stations, and so on. In this application, basically the base stations periodically receive the information from the sensors and other devices (e.g., the location of cars, information about crashes, traffic congestion or weather conditions). With this information, the base stations calculate the recommended speed for each vehicle on a certain stretch of road and send it to the vehicles (through the RSUs to the vehicle OBUs or to other user devices, such as smartphones).

In the CSA scenario, avoiding malicious attacks is extremely important. For example, to indicate that the recommended speed is 100 kph when you have traffic congestion in the vicinity could cause an accident. Therefore, the messages sent between the nodes and the base station must be properly encrypted, and all of the nodes must be trustworthy. Therefore, the self-protection of the whole system is required. However, very hard security policies have a penalty in efficiency, especially in energy and performance, two typical requirements of these applications. Therefore, the security policy to be applied in each situation must be negotiated to make a trade-off between the necessary security and the efficiency. Additionally, as these situations can change quickly, these policies have to be (re)negotiated. For instance, in the case where malicious software is suspected in a given WSN (detected by intruder detection algorithms [17]), the security needs to be increased, and the suspect nodes must be isolated. To the contrary, on roads where the main interest is to preserve the lifetime of the system and there is no risk of malicious attacks, the security can be a little bit weaker. Similarly, in situations where communication performance is required (e.g., high traffic density), the security could also be relaxed. On the other hand, in exceptional circumstances, the interception of data sent by certain vehicles (e.g., a presidential motorcade) has to be totally avoided. In this case, the security policy applied to these specific nodes must be modified. Furthermore, other special situations, such as emergencies or attempted terrorist attacks, require another kind of global security and/or intrusion detection.

## 3. Challenges and Our Approach

In this section we describe, in more detail, the main challenges that need to be taken into account in order to add security to WSNs and to dynamically (re)configure the security level at runtime. We also indicate how our approach copes with these challenges.

Challenge1: Build secure applications that run on heterogeneous nodes with limited capabilities. The nodes of the WSNs are heterogeneous, and thus, the security solution deployed at each node needs to be adapted to the characteristics of every node. Furthermore, these nodes have limited capabilities, and the security solutions need to be carefully specified to avoid consuming too many resources. This means that we need to define a family of security solutions, providing different implementations for the different nodes in the WSN, while also taking into account the constraints on the resources available for each node. Our approach: Using the software product line mechanisms, already existing in the development process defined as part of FamiWare, we will be able to generate customized instances of the FamiWare middleware, each one ready to be run on a specific node of the WSN. In this way, every instance of FamiWare has just the security services or algorithms required by the applications installed in each node. Therefore, we avoid loading a big chunk of security code into the limited tiny devices. We will integrate the new security components defined by INTER-TRUST as part of the FamiWare product line architecture.

Challenge2: Dynamic negotiation of security policies for WSNs. The conditions of the running environment of the WSNs applications could change. Then, the security policies may need to be adapted to the new conditions. This adaptation requires a previous negotiation between the different parts of the application. Our approach: As previously said, INTER-TRUST is the only framework that provides dynamic negotiation of security policies. By integrating the INTER-TRUST framework with FamiWare, we provide a middleware that allows the dynamic negotiation of the security policies for the sensor nodes already included as part of FamiWare.

Challenge3: Monitor the changes in the context. These changes can be general (e.g., changes in the amount of memory or the battery level available) or can be specific to security. In order to react to these changes, the environment needs to be monitored. Our approach: Both INTER-TRUST framework and FamiWare middleware include specific components for monitoring the environment. Therefore, we will add those security components provided by INTER-TRUST that are not present in FamiWare. The main contribution of the process proposed here is to guarantee that among all of the existing monitoring components, only the most necessary and appropriate ones are incorporated into the solution for each sensor node. This is done via the configuration process provided by FamiWare.

Challenge4: Define a dynamic reconfiguration service to endow applications with self-protection. When the environment or the security requirements change at runtime, the system must be self-protected to maintain a proper and secure functioning. Our approach: Firstly, the new security policies to be used need to be negotiated using INTER-TRUST. Secondly, the new security policy needs to be analyzed to determine whether the changes to the security policy imply changes in the system's components. If changes in the system's components are required, then a reconfiguration plan needs to be generated to carry out these changes without stopping or crashing the system. The last two tasks are performed by FamiWare. These plans contain the implementation level activities to be carried out in order to instantiate the new security policies.

Challenge5: Provide an efficient solution able to run on a WSN. As WSNs are composed of a large number of tiny devices, the self-protection mechanism should consume the least number of resources as possible. Our approach: The FamiWare reconfiguration mechanism pays special attention to efficiency, as we will show in the evaluation (see Section 6). We focus on proving that resource consumption of this mechanism is optimal for a typical sensor type (MICAz). We also check that our solution is scalable to the number of nodes. Furthermore, we demonstrate that the overhead and latency of the self-adaptation is not critical compared with the benefits of having secure applications in WSNs.

## 4. Integration of FamiWare and INTER-TRUST

**Figure 4.**
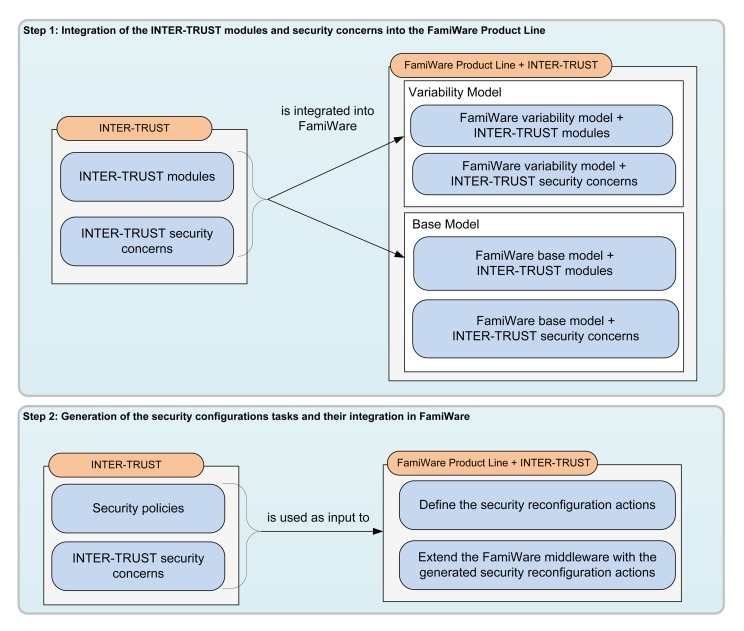
Steps for the integration of INTER-TRUST into the FamiWare family of middlewares.

As explained, FamiWare is a family of middlewares specially designed and implemented for dealing with the heterogeneity of WSNs devices. It also provides a mechanism to be reconfigured at runtime when the context changes. However, it does not focus on providing security. Therefore, if we want to extend FamiWare with the benefits provided by the INTER-TRUST framework, the first thing to do is to integrate INTER-TRUST into the FamiWare family. As shown in Figure 4, this implies several tasks that have been organized into two main steps. The first step consists of the integration of the INTER-TRUST modules and the INTER-TRUST security concerns into the FamiWare product line. This implies the extension of both the variability model and the base model of FamiWare. This step allows the generation of different FamiWare middleware configurations, customized for the necessities of each device, including the customization of the security modules and concerns. In the second step, the security policies specified in INTER-TRUST and the security concerns (to be deployed for enforcing those security policies) are taken as input to define and implement the reconfiguration actions. These reconfiguration actions need to be executed in order to generate a new configuration adapted to the new security context. Therefore, these actions must be part of the reconfiguration plans that FamiWare generates at the implementation level to switch from the current FamiWare configuration to the next one. This step enables the security requirements to be adapted at runtime.

In the rest of this section, we explain these steps in more detail. The first step is detailed in Section 4.1 and the second step in Section 4.2. Finally, Section 4.3 shows different configurations of the FamiWare middleware with security for different devices of our ITS case study.

### 4.1. Integration of the INTER-TRUST Modules and Security Concerns

This section is organized in two main subsections. Section 4.1.1 principally focuses on the integration of the INTER-TRUST modules, while Section 4.1.2 focuses on the integration of the INTER-TRUST security concerns.

#### 4.1.1. Integration of the INTER-TRUST Modules

**Figure 5.**
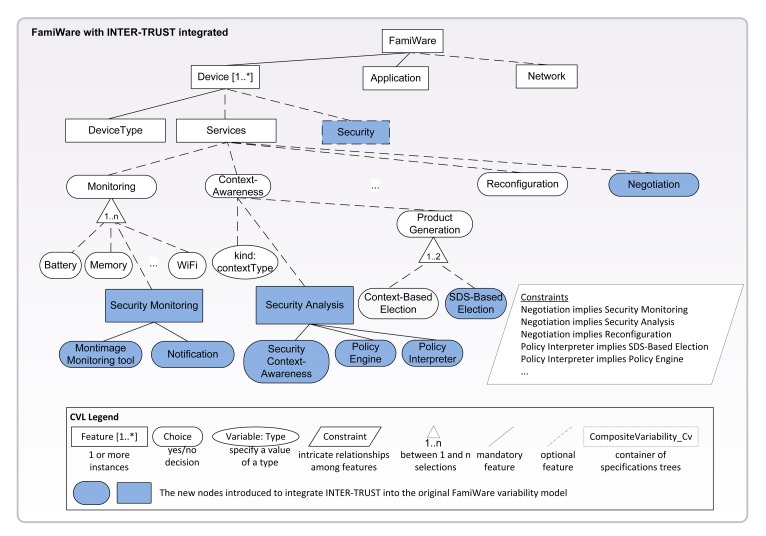
FamiWare + INTER-TRUST variability model.

In Section 2.1, we described how a different configuration of the FamiWare middleware could be deployed in each device according to that device's characteristics, the applications that this device runs and the network used to connect several devices. Now, with the integration of the INTER-TRUST framework into the FamiWare middleware, the security also has to be taken into account to configure the FamiWare middleware. To do so, as shown in Figure 5, the VSpec tree of FamiWare is extended with the security feature. Due to the correspondence between feature models [18] and CVL VSpec trees, for simplicity, we use the term ‘feature’ throughout the paper to refer to the term ‘VSpec tree node’. Since the security concerns instantiated could be different for each device, the security feature has been included as a child of the device feature instead of defining it as a child of the FamiWare feature. This feature is a CVL composite variability. This means that it is a container of another VSpec tree describing the variability of all of the security concerns that could be instantiated in each device (this is described in Section 4.1.2).

In addition to the security concerns, the INTER-TRUST modules also need to be incorporated as part of the services provided by FamiWare. Thus, we need to extend these services for the special requirements of the INTER-TRUST dynamic negotiation of security policies. On the one hand, FamiWare does not provide any service for negotiation. Thus, we need to extend the variability model of FamiWare with a completely new service, the negotiation service, which is included at the same level as the monitoring, context awareness and reconfiguration services. On the other hand, the already existing FamiWare services need to be extended to include the monitoring, context awareness and reconfiguration features related to security that are specific to INTER-TRUST:

Monitoring: This is represented in the FamiWare VSpec tree as an optional element with one child or more for each context that can be monitored by the middleware. Therefore, as we need to monitor the issues related to security (e.g., vulnerabilities, security algorithm overheads), we have to add a new element to the children of the monitoring element called security monitoring in Figure 5. This variable element has two children, the Montimage Monitoring Tool and the notification features. They are described in Section 2.2 and are the modules and tools used in the INTER-TRUST framework to monitor the application's security.
Context awareness: The context awareness feature of FamiWare is represented by two elements. One is the kind of context of which a certain application is required to take care. The other is the list of changes to be executed to generate a new product when a context modification implies the system's reconfiguration. In INTER-TRUST, this is carried out by the security context awareness together with the policy interpreter and the policy engine modules. These modules decide whether or not a new security policy must be negotiated and the changes to be performed in the system security after the negotiation. Thus, a new feature called security analysis has been added as a child of the context awareness feature of FamiWare that includes the security context awareness, policy engine and policy interpreter children. Moreover, a new feature has been added as a child of the product generation feature to indicate that, in the case of security, the new product configuration needs to be generated, taking into account the information dynamically generated by the policy interpreter module of the INTER-TRUST framework. Thus, now, the reconfiguration plans could be generated using different processes: the context-based election feature represents the original process used by FamiWare; the Security Deployment Specification (SDS)-based election feature represents the process that generates the reconfiguration plans taking as input the information generated by the policy interpreter module of INTER-TRUST. The SDS [19] is a custom format defined by INTER-TRUST to indicate the security concerns that need to be deployed for each security policy and the security functionalities that need to be provided by each concern (e.g., the authentication concern with a user-password functionality). In both cases, a reconfiguration plan is generated at runtime by FamiWare.
Reconfiguration: In FamiWare, this service reconfigures the nodes of the WSN by executing the corresponding reconfiguration plan in each node of the network. With the integration of INTER-TRUST, this module is also in charge of reconfiguring the security of the system by instantiating the new negotiated security policy in all of the necessary sensor nodes of the network. Since the context awareness module generates an reconfiguration plan with the same format for both generic and security-specific reconfigurations, the original reconfiguration service of FamiWare does not need to be extended and can be used as is to reconfigure the security of the FamiWare applications. However, the reconfiguration tasks for security need to be defined and implemented as explained in the following subsection.

The main advantage of modeling the security concerns and the modules of INTER-TRUST as variable characteristics of the FamiWare product line is that it is possible to generate different middleware configurations with different security policies for the heterogeneous devices of a WSN. Furthermore, using the variability model at runtime, it is possible to renegotiate these security policies at runtime.

As said, in CVL, the OCL constraints specify the dependencies between the aforementioned features. In addition to the constraints already specified for the FamiWare variability model, Figure 5 shows part of the constraints for the new features. For instance, the constraints of the figure specify that the selection of the negotiation feature implies the selection of the security monitoring and the security analysis features or that the selection of the policy interpreter feature implies the selection of the SDS-based election feature.

Note that we have focused on showing how the variability model of FamiWare is extended because we consider that this is the most interesting part of Step 1 shown in Figure 4. The base model is also modified by adapting the implementation of the INTER-TRUST modules to the event-based component model of FamiWare and by including the new components in the software architecture of FamiWare

#### 4.1.2. Integration of the Security Concerns

**Figure 6.**
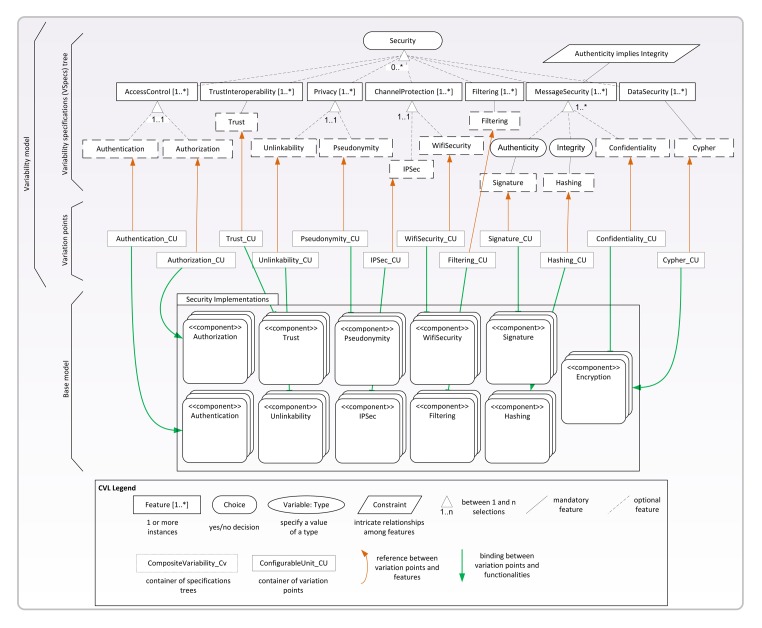
Composite variability: security concerns.

**Figure 7.**
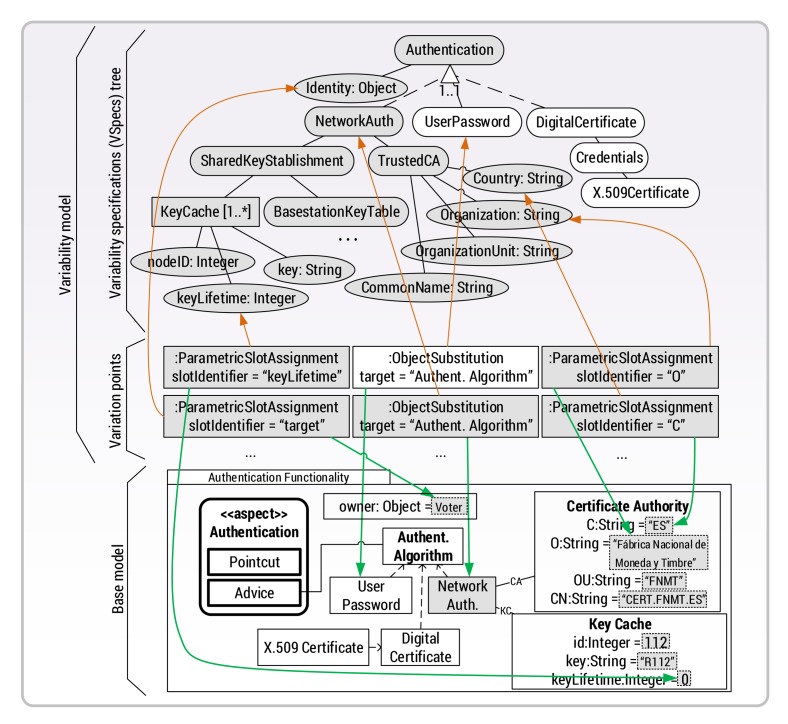
Resolution model of the authentication concern.

Figure 6 shows the VSpec tree that models the variability of the INTER-TRUST security concerns (*i.e.*, the VSpec tree contained in the security composite variability shown in Figure 5). The top of Figure 6 shows the abstract part in which all of the security concerns, functionalities, attributes and parameters that can be reconfigured at runtime are specified (*i.e.*, the variability specification tree for security). The bottom of Figure 6 shows a representation of the specific implementation of the security functionalities encapsulated in the FamiWare components (*i.e.*, the base model of the security concerns). Details of the parameters of each security concern in the variability specifications tree and in the FamiWare components are hidden in Figure 6 to simplify it. Some of these details are shown in Figure 7 for the authentication functionality and are detailed below. Finally, the security features of the tree and the specific functionality of the FamiWare components for security are linked by using the CVL variation points (middle of Figure 6). A particular selection of the features in the tree defines a particular configuration of the security components.

**Figure 8.**
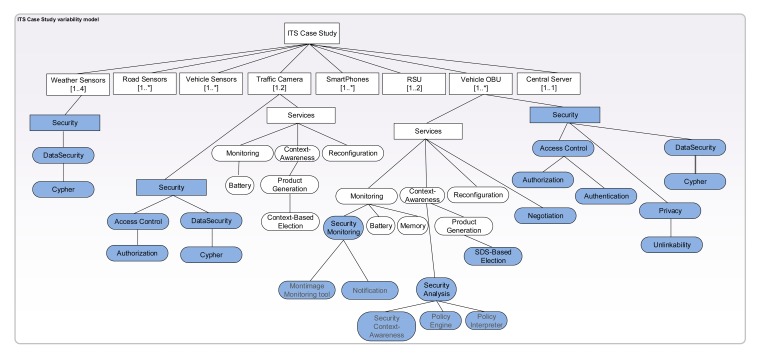
Intelligent transport system (ITS) case study variability model.

As stated, Figure 7 shows the kinds of details that were omitted for the security concerns in Figure 6. In particular, the required configuration for the attributes and parameters of the authentication concern are shown. The graphical elements shown in grey indicate those elements selected in a particular configuration. In this case, the VSpec NetworkAuthhas been selected to indicate that a dynamic authentication and key establishment protocol [5] will be used as the authentication mechanism. Moreover, the configuration also includes the parameters for the certificate authority (TrustedCA) such as the information about the organization that issued the certificate and the parameters for the key caches that will store the session keys to be used with the certificate (nodeID, Keyand KeyLifetime). Note that this is only an example, and the variability model allows different instances of each component modeling a security concern to be created and configured. Moreover, as illustrated in Figure 8 in Section 4.3, the variability model also allows the use of different authentication mechanisms for different parts of the same application.

Finally, the FamiWare base model is extended to integrate the security aspects provided by INTER-TRUST. Security concerns are implemented in INTER-TRUST using the aspect-oriented programming approach [20]. However, the approach presented in this paper is not affected by the use or not of aspects. FamiWare components communicate using a publish/subscribe interaction model. This means that in order to integrate the security aspects provided by INTER-TRUST, we need to define a FamiWare wrapper component that wraps the behavior provided by the advice of the INTER-TRUST aspects. This is straightforward, since the aspects in INTER-TRUST act also as a wrapper of the security behavior, which is implemented independently of a particular technology.

### 4.2. Generation and Integration of the Security Reconfiguration Actions into FamiWare

In order to maintain the correspondence between the running code and the model, FamiWare defines a two-level mapping between the modifications of the features in the VSpec tree and the implementation level tasks [21] to be executed for the system reconfiguration.

The first mapping is a table that specifies for each feature of the VSpec tree how the presence/absence of that feature implies the execution of a set of generic reconfiguration tasks. Examples of these generic tasks are activating or deactivating a sensor, changing the role played by a sensor, adding a service to a certain FamiWare middleware instantiation, removing a running service or changing the configuration of a service. Since these tasks are generic, they are already provided by FamiWare and are directly reusable in the extension of FamiWare with INTER-TRUST. An example of this correspondence would be: “when the cypher feature was not selected in the running product and is now selected in the VSpec tree representing the adapted product, a reconfiguration task to add the service for encrypt/decrypt messages has to be executed”. At the implementation level, this corresponds to the task *AddService*(*Device_n_*, *Service_m_*) with two input parameters (the device identification and the service name). Then, the tasks can have input parameters; thus, we define a task as *t*(*p*_1_, …, *p_n_*), where *n* is the number of parameters, which can be variable. A FamiWare reconfiguration plan is a sequence of tasks specified in OWL-S [22], an ontology of services for semantic descriptions of web services. For instance, examples of tasks, in this reduced version of OWL-S, to add the encryption service to a traffic camera and to configure it with a particular encryption algorithm, would be like this:
<AtomicProcess ID=“AddService”> <Input ID=“TrafficCamera2”/> <Input ID=“Encryption”/></AtomicProcess><AtomicProcess ID=“ConfigureService”> <Input ID=“Encryption”/> <Input ID=“encryptAlg2”/></AtomicProcess>

Using these generic reconfiguration tasks, FamiWare permits the reconfiguration of both the hardware of each device and its internal FamiWare architecture. Regarding hardware reconfiguration, FamiWare could, for instance, switch the radio chip of the sensor to on/off or change the whole state of the sensor. With regards to the modifications in the software architecture, it is possible to perform coarse-grained modifications, such as remove or add a service, and it is also possible to make fine-grained modifications, like changing some parameters of a service.

The second mapping specifies how these generic reconfiguration tasks are implemented for each device, application and/or new service. Since the implementation may be different for each device, application and/or service, a list of specific reconfiguration actions need to be implemented for each generic task. Some actions are specific to a concrete type of device, as is the case of putting to sleep a sensor device (*i.e.*, switching off a MICAz mote), or to a concrete application, such as the specific places in the application where a security concern needs to be ensured (e.g., authentication must be ensured when the vehicle interacts with the server for the first time).

The current implementation of FamiWare already provides the code of the device-specific actions for a broad range of sensors/devices and platforms (e.g., TinyOS, SunSPOT). However, in the case of the reconfiguration actions that are specific to a certain application or to a new service (e.g., security), the code of the particular actions to be executed for reconfiguring the systems must be manually provided to FamiWare. Thus, according to the second step shown in Figure 4, the integration of INTER-TRUST into FamiWare requires that the FamiWare variability models of Figures 5 and 6 are complemented with the list of security reconfiguration actions that need to be executed to reconfigure the application security level at runtime. Furthermore, the mapping between the generic reconfiguration tasks and these security-specific actions needs to be defined for each security concern and security policy. This means that, in order to provide security in FamiWare, we need to implement these basic actions for activating each security concern (e.g., encryption) that have been previously incorporated in the security feature of the FamiWare VSpec tree. For instance, in the case of adding the cypher service using encryption algorithm, encryptAlg2, we need an implementation action to add the component that implements the encryption algorithm. Following the publish and subscribe model of FamiWare, the code to add the service to encrypt messages in a sensor/device would be the following:
addComponents(<sensorID>, encryptAlg2)publisher(<sensorID>, encrypted)subscriber(<sensorID>, encrypted)

This means that, now, Traffic Camera 2 publishes/receives encrypted messages. Note that this is done once, and then, after the action has been implemented and incorporated inside FamiWare, it will be used each time an application decides to use the extension of FamiWare with INTER-TRUST. There are other actions, such as an action to connect the application components with the encryption component in the places where encryption must be ensured, that will be specific for each application. Therefore, we must provide the code that implements the security policies that are going to be considered in a certain application, the code for activating or instantiating them and the correspondence between the code and the implementation level tasks.

After implementing all of the required specific implementation tasks, FamiWare automatically generates at runtime the reconfiguration plans with the specific implementation level tasks necessary to adapt the system. In the next section, we show how FamiWare uses the implementation tasks and the correspondence between features and implementation tasks to generate, at runtime, the reconfiguration plans that need to be executed in order to reconfigure the system for security issues.

### 4.3. FamiWare with Security for the ITS Case Study

Now that we have integrated FamiWare and INTER-TRUST, we can generate a VSpec tree configuration of the FamiWare family for the different nodes of our ITS case study (*i.e.*, the vehicle sensors and devices, the road sensors, the RSU devices and the central server). Focusing on security, each device in the ITS case study has different necessities with regard to its security level. They also have different necessities regarding the reconfiguration of the security at that device.

Figure 8 shows a configuration of the VSpec tree for our case study. It is not complete so as to focus only on showing the different security levels that can be configured for each node of the network. Concretely, we have provided details for three different kinds of devices. An example of the first type of device is the weather sensors at the left-hand side of Figure 8. These sensors are highly constrained devices installed in the roads in order to obtain precise information about the weather conditions. This information allows the adaptation of the speed to the changes in the weather. The information provided by these sensors is critical from a security point of view, since an intruder could change it to indicate a sunny day when really it is raining, causing an accident if the speed limit is increased in bad weather conditions. This could be avoided by cyphering the information sent by the weather sensors as is shown in Figure 8, where the security/DataSecurity/cypher features have been selected. However, no reconfiguration support of any kind is going to be provided for these devices, and thus, the FamiWare services for reconfiguration were not selected.

An example of the second type of device is the traffic cameras. These are also constrained devices where the security is critical. First, only authorized users will receive the information transmitted by the cameras. Second, all of the information transmitted by the cameras must be encrypted. In order to deploy the required security level in the traffic cameras, the access control/authorization and the DataSecurity/cypher features need to be selected for this specific configuration. Note that only four features from the security variability model defined in Figure 6 have been selected to configure the security level of the traffic cameras. The application developer has considered that in order to avoid the consumption of battery and memory in traffic cameras, these devices will be deployed with an initial security configuration that will not be changed at runtime. Thus, among the services deployed for these devices, the INTER-TRUST modules will not be deployed. However, the difference with the weather sensors is that, in the traffic cameras, other resources, such as the battery, can be monitored (by the monitoring service) and could be reconfigured at runtime (by the reconfiguration service) according to the contextual information received (by the context awareness service).

Finally, an example of the third type of device is the vehicle's OBU. These devices have more resources, and thus, a security solution that can be dynamically reconfigured at runtime can be deployed with fewer limitations on the consumed resources. Thus, in Figure 8, we can see how, under the services of the OBUs, all of the modules provided by INTER-TRUST in order to negotiate and reconfigure the security level of applications will be deployed. Moreover, a large set of security concerns, including authentication, authorization, encryption of data and privacy, are deployed in these nodes.

## 5. Dynamic Reconfiguration of Security in FamiWare

FamiWare also uses the VSpec tree described in the previous sections at runtime to drive the reconfiguration of the middleware with self-adaptation purposes. Concretely, FamiWare uses a models@run.time [23] approach to generate and to interpret the reconfiguration plans with the tasks to execute for switching from a configuration of the middleware to a new configuration in response to context changes. By using a models@run.time approach, the changes are performed at the architectural level, and thus, the architectural configurations running in each network device, and at all times, are correct according to the global network and system restrictions. Other approaches implement reconfiguration mechanisms at the code level [24,25], but the main drawback is that everything is hard-coded, so the correspondence between the reconfigured code and the models used at design time is lost. This makes it impossible to ensure that each middleware instance will enter a valid state after a reconfiguration is executed in the whole system.

**Figure 9.**
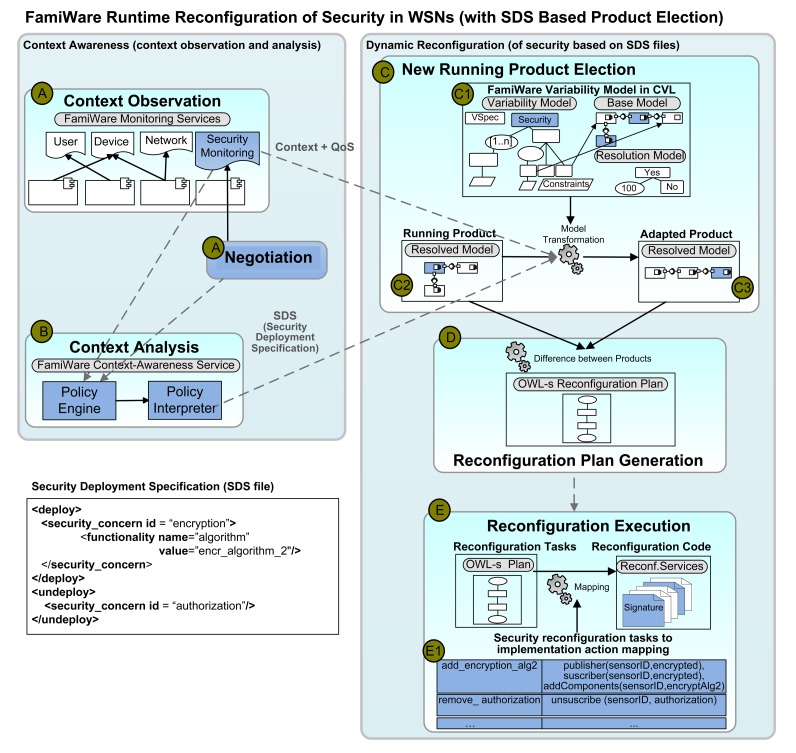
FamiWare runtime reconfiguration of security.

Following an autonomic computing approach [26], the monitoring, context awareness and reconfiguration services, described in the previous section, collaborate in order to reconfigure the middleware at runtime. In Figure 9, we focus on the reconfiguration of security, but a similar process is followed to reconfigure any other functionality in the WSN. The only difference is the use of the INTER-TRUST modules that have been specifically introduced to manage security in FamiWare.

Concretely, Figure 9 represents the process followed when the SDS-based election feature is selected from the VSpec tree shown in Figure 5. Steps A and B represent the context observation and context analysis tasks, while Steps C, D and E represent the dynamic reconfiguration of security in FamiWare. In Step A, we see how the reconfiguration of security can occur due to the monitoring of the security contexts previously modeled or by a renegotiation of the security policies using the INTER-TRUST negotiation service. When this occurs, the changes need to be analyzed (Step B) to decide whether or not the middleware self-adaptation is required. Using the models@runtime approach provided by FamiWare (C1), this self-adaptation (self-protection when we talk about security) implies: (1) the generation of a new reconfiguration plan (Step D) using the models available at runtime (C1 and C2), the contextual information provided by Step A and the SDS file generated by the policy interpreter module in Step B; and (2) the execution of the reconfiguration plan (Step E) to adapt the middleware services (security in this case).

The reconfiguration plans are calculated by applying a difference operator between two configurations/products, the active one (C2) and the new configuration to be deployed (C3). The plans are carried out to guarantee that the system functions after a context variation, and the main goal is to change from a VSpec tree configuration to another valid configuration. The tasks are performed both at the VSpec tree level and at the implementation level with a semantic correspondence between them. As described in the previous section, at the implementation level, a task could be: modifying the parameter of a component, connecting two components, removing a component or sleeping sensor devices, *etc*. At the VSpec tree level, every task is interpreted as either a feature addition, a feature removal or a feature modification. The changes can be coarse grained (e.g., adding a new middleware component as, for instance, a new authentication component) or fine grained (e.g., modifying a parameter of an existing middleware component, such as, for instance, changing the certificate entity used by an existing authentication component).

FamiWare allows the generation of plans both at design and at runtime. In the case of the security reconfiguration, the plans need to be generated at runtime, because the information to go from the existing configuration to a new one is dynamically generated by the policy engine + policy interpreter modules. This information is in the SDS file that is used to transform the VSpec tree of the running product to the VSpec tree of the adapted produced, as shown in Figure 9.

In that SDS file, we see how the output of the policy interpreter indicates that the encryption concern using the encr_algorith_2need to be deployed and that the authorization concern must be undeployed from the system. Using this SDS file, the running product (C2) and the variability model (C1), FamiWare automatically obtains the configuration that represents the adapted product (C3). In this configuration, the DataSecurity and cypher features will be selected and the authorization feature unselected. Then, using the difference operator, a reconfiguration plan with two tasks (one to add the encryption components and another to remove the authorization ones in all sensors of the WSN) is automatically generated:
<Sequence> <AtomicProcess ID= “AddService”>  <Input ID= “AllSensors”/>  <Input ID= “Encryption”/> <AtomicProcess ID= “RemoveService”>  <Input ID= “AllSensors”/>  <Input ID= “Authorization”/></Sequence>

As we explained in the previous section, this plan is interpreted by the FamiWare reconfiguration service deployed in each WSN node. The specific reconfiguration code for reconfiguring the security policies must be provided, so we use the mapping defined in the previous section between the security reconfiguration tasks and the specific implementation actions (label E1 in Figure 9), which needs to be executed for the system reconfiguration. In the mapping of the example, we can observe that for adding an encryption algorithm to a sensor, as FamiWare follows a publish/subscribe architecture, we need to execute the following actions: (1) from now on, this sensor will publish its data under the encrypted topic; (2) this sensor is now subscribed to the encrypted topic (*i.e.*, it will receive encrypted messages); (3) the components that implement the selected encryption algorithm have to be added to this sensor. In a similar way, to remove the authorization, this sensor has to unsubscribe from this topic.

## 6. Evaluation

The evaluation of the approach presented in this paper has been organized into two subsections. In the first subsection, some quantitative experimental results are shown. Then, in the second subsection, we discuss both the benefits and the main limitations of our approach.

### 6.1. Experimental Results

The goal of the experiments presented is three-fold: (1) they quantify the memory footprint used and the spent battery by the new security self-adaptation (*i.e.*, self-protection) mechanism of FamiWare; (2) they evaluate the scalability of FamiWare, regarding the number of security policies and reconfiguration plans; and (3) they calculate the overhead produced by the security self-adaptation mechanism, measuring the latency, since the new configuration model has been generated and the reconfiguration has been accomplished. For the experiments, we have measured the FamiWare implementation realized for MICAz motes with TinyOS [27].

Resource consumption: Since FamiWare is composed of a variable set of services (including the new security and reconfiguration modules, among others), the memory usage on a sensor depends on how many services are in the current configuration and on the size of each one. Furthermore, since any TinyOS application is compiled together with the OS in one image, we also have to include the OS as part of our measures. Specifically, to perform the reconfiguration mechanism of the security policies, apart from a WSN routing protocol to transmit data, the minimal instantiation must have one security monitoring service, the context awareness service and the reconfiguration service plus a simple security module. Therefore, in this case, using, for instance, the Drip [28] routing protocol, the memory footprint is greatly reduced. Considering that typical MICAz nodes have 4 KB of RAM, this instantiation consumes 51% (21,340 bytes) of RAM. On the other hand, the maximum architectural configuration for reconfiguring the middleware, considering all of the security monitoring services to observe different kinds of security contexts, the microkernel, Drip protocol and a simple application (123 bytes) and several security modules, uses 80% (3400 bytes) of the memory, so there is still 20% free for other services and applications. Note that this maximum configuration, which includes services to monitor the global status of a part of the network, is only instantiated in cluster heads, which are usually device sensors with higher capacities than the regular MICAz.

With respect to the evaluation of the energy consumption of the security dynamic reconfiguration of FamiWare, we have measured the battery waste of such process in a WSN. The battery capacity of the MICAz motes is 2000 mAh and lasts about a year. We have simulated several reconfigurations of a 20-node network using security reconfiguration plans similar to the ones defined in the previous sections (Sections 4 and 5), when the energy of the nodes is around 30% (600 mAh) on average. We compare these results with the energy expenditure of the same system, but without reconfiguration and, therefore, maintaining the same security policy defined at design time. The average of the network residual energy starts to decrease when the reconfiguration is being performed (simulation interval from 0 to 1000 s). This is due to the extra cost of operations for reconfiguring the nodes and the sink. For instance, they must be in the “active” state with the radio chip “on”, and they must access their flash memory. During the reconfiguration, the remaining energy average drops to 59,984 mAh for the first 1000 s simulated, while in the system without reconfiguration, it only drops to 599,911 mAh. There is a difference of 0.071 mAh, which is the price of reconfiguration in terms of energy, but after it finishes, the energy expenditure is more or less equal for the reconfigured system as for the static one (depending on the security policy installed, since some of them consume more energy than others). This amount of spent battery (0.071 mAh) is similar to the amount that the same WSN wastes when the sensors are monitoring several issues (e.g., temperature and humidity) with 1 s of frequency, and they spread this sensed information throughout the network. Therefore, we consider that this extra waste of energy is acceptable for these long-lived systems, considering that they will provide more flexible security levels than static systems.

Summarizing, the resource consumption of FamiWare for the security dynamic reconfiguration is satisfactory and highly suited to the memory and battery constraints of real sensors.

**Figure 10.**
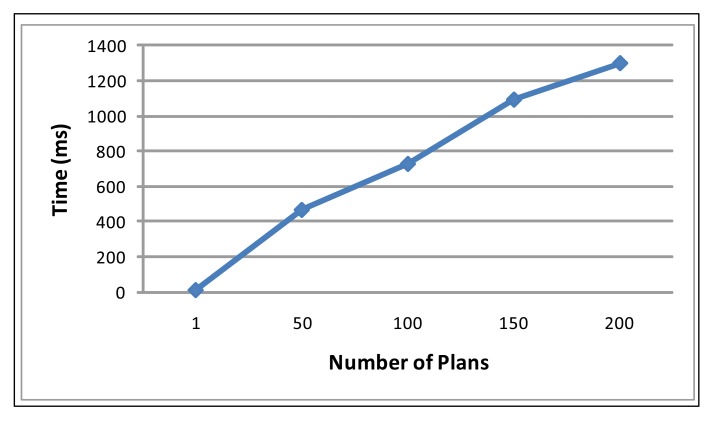
Time that FamiWare takes for finding and reading a plan.

Scalability: We assess the scalability performance in terms of the number of plans that an ordinary node can load in its flash memory. This will be related to the number of different security policies that can be managed, since in the security context, we need a different plan to change from one security policy to another one. MICAz motes have 512 KB of flash, 384 KB of which are used for the routing protocol and for loading the binary code of the new images [27]. Therefore, if we reserve 98 KB for loading the plans, there is still 30 KB free, enough for applications or sensed data. The size average for the plans (after unnecessary tags of the OWL-S file are removed) is about 500 bytes, so there is enough space to load 200 plans, this is the upper limit of the number of plans. On the other hand, in an ordinary node (not sink or cluster-head), we can reduce the space reserved for plans, since these nodes do not need this high number of plans loaded inside the memory. The time needed to search a plan in the flash memory increases when there are numerous plans loaded. As Figure 10 depicts, the increase of time is linear with respect to the number of plans. Finally, the maximum time needed to find a plan, if we consider 200 plans loaded, is 1298 ms, an insignificant delay, compared with other sensor tasks.

Considering that we need a plan to switch from one security policy to another, FamiWare works well with 14 different security policies, since, to quickly manage 14 policies, we need to have 14 × 14 = 196 plans loaded in the sensors. In the case that we need to manage more policies, we can load new plans on the fly. This increases the reconfiguration time due to communication delay, as we see in the latency evaluation.

Overhead and Latency: As for the negotiation, the generation of the new configuration and the selection of the plan are all done outside the sensor devices; we just measure the interval time from when the reconfiguration service receives the plan to change the security policy until the reconfiguration has been completed in all of the nodes. The average time taken by the reconfiguration service in different security policy modifications is 570 m. The longest time in this service corresponds to the execution of the tasks that comprise the plans. This is because many tasks implies the reconfiguration of several remote nodes. Therefore, this entails having to send several messages to different nodes, and the time this takes depends on the routing protocol used. For instance, using Drip protocol in one hop (without intermediate nodes), it takes 8 ms, but if we consider three intermediate nodes, this time increases up to 6.43 s. It is worth noting that if we increase the number of sensor nodes, but they are still one hop from the sink, the time needed to reconfigure them does not increase significantly. Nevertheless, if the sink has to use some intermediate nodes to reconfigure other nodes, the time greatly increases, but it depends on the structure of the network and the routing protocol used, as in other WSN platforms. Therefore, the overhead produced to reconfigure the system is insignificant when compared with the necessary time to send a packet.

#### 6.2. Discussion

Analyzing the overhead, latency and resource consumption, we can conclude that the adaptation of the security policies to the contexts has an extra cost (especially in tiny devices like sensors), but in return, we obtain secure applications optimized for the different situations. This implies that the applications will be more secure and will consume only the necessary resources. Nevertheless, in cases of critical situations, such as emergency scenarios, where the time response has to be very short at a certain moment, the reconfiguration must be postponed if and when the instantiating of the adapted security policies would not be strictly necessary for the correct functioning of the system. Therefore, in these cases, we must avoid the reconfiguration if it is only being done for the optimization of the security, especially if this reconfiguration implies having to self-adapt many devices and takes a long time. We therefore need to realize some trade-offs, taking into account the expected response time, the security needs and the reconfiguration overhead, to decide when the reconfiguration must be carried out.

On the other hand, using models@run.time, the product running in each device corresponds to the resolved model that satisfies each context. Then, as we have generated them, respecting the constraints between the variable elements, we can ensure that the global system enters a valid state after the reconfiguration. Furthermore, as FamiWare defines the dependencies between the different sensor nodes as constraints between clones in the variability model, the running product after the reconfiguration maintains the compatibility between the devices. Nonetheless, the only models that are loaded in the sensors and sent over the network are the reconfiguration plans that consist of small files (around 500 bytes), twenty times smaller in size than the variability model.

Therefore, we ensure the correct functioning of the system after the reconfiguration if the security policies and reconfiguration task implementations respect the models. However, thus far, we have not monitored this reconfiguration process, nor have we observed that the system works as expected at runtime. This is done in INTER-TRUST using both active testing and fuzz testing techniques [19], where these modules verify, before and during the operational phases, that the negotiated contracts are respected and that the security policies are respected in dynamic systems. However, these modules of INTER-TRUST have not been integrated into FamiWare, yet.

Regarding the necessity of the evolution of these kinds of systems where new technologies are appearing everyday, using the models@run.time and the SPL paradigm to model the security, the addition of new security policies (or the modification of existing ones) is very easy. As the security concerns are modeled in the VSpec tree shown in Figure 6, the new security policy has to be added to this composite variability, as well as the correspondent modules in the base model. FamiWare will then be in charge of reconfiguring the system for this new security policy in the same way as it did for the rest of the policies. Therefore, we can say that our process is prepared for the evolution. However, this evolution is not totally automatic, because the implementation code of the new policy and the specific reconfiguration actions has to be manually provided to FamiWare.

Finally, we have tested our reconfiguration process for the ITS case study that has been previously used to validate both INTER-TRUST and FamiWare. However, INTER-TRUST has also been tested in an e-voting scenario, and FamiWare has been implemented for ambient assisted living applications. Therefore, it would be useful to also test our security reconfiguration process in both case studies.

## 7. Related Work

The Related Work section is organized into two subsections. The first subsection discusses those approaches that provide support for the dynamic adaptation of security, in comparison with the INTER-TRUST framework. The second subsection discusses the main characteristics of existing DSPL approaches, in comparison with the reconfiguration provided by FamiWare middleware.

### 7.1. Dynamic Adaptation of Security

There are a lot of approaches that try to deal with runtime adaptation of security. For instance, in [29], the authors present a policy-based approach for automating the integration of security mechanisms into Java-based business applications. They use security@runtime, a Domain-Specific Language (DSL), for the specification of security configurations based on authorization, obligation and reaction policies. The approach presented in this paper, in contrast, is suitable for using security policies specified in any model (e.g., OrBAC—Organization-Based Access Control), since the mapping between the policies and the security functionalities is made at an abstract level of the variability model. Another difference with the approach presented in this paper is that we separate the monitoring of changes in the application and the integration of the security functionality following the MAPE-K (Monitoring, Analysis, Plan, Execution—Knowledge) loop, while they integrate the security functionalities in the same monitoring events. Moreover, they implement the security rules in separate classes, but this code is application dependent, while in our approach, the security rules do not need to be hard-coded, improving the evolution of the policies.

In [30], the authors use policy-based security profiles to make logical and knowledge-based decisions within open service environments, and it uses a layered holistic model [31] to describe security (*i.e.*, security requirements are defined using security profiles that describe the interlinking of security policies to instances of services). However, in our approach, the security policies are decoupled from the specific knowledge of the application and from the implementation of the security functionality in various aspects, and this improves the reuse of both the security policies and the security functionalities.

Model-driven security (MDS) is often used to adapt security dynamically following different approaches: UMLSec (UML for secure systems) [32], SecureUML (access control language based on UML) [33], OpenPMF (Open Policy Management Framework) [34,35], SECTET (framework for model-driven security) [36], *etc*. For instance, in [37], models@runtime is used to keep an architectural model synchronized with a policy, but this approach only supports access control policies and not any kind of security functionality, as does our security adaptation service. This is a general limitation in many security policy-based approaches, because they are primarily focused only on access control or authorization concerns [34,35,38,39] or they are focused only on a specific domain, such as mobile cloud [40] or service-oriented architecture (SOA) [34–36,38].

There are also some generic approaches for reconfiguration at runtime that do not only concentrate on security concerns. In [41], Gamez *et al.* propose a reconfiguration mechanism that switches between different architectural configurations at run-time. The configurations are based on the specialization of feature models, and the reconfiguration plans are automatically generated from the differences between them. They propagate changes in configurations at the architectural level instead of the direct aspect implementation, as we do.

### 7.2. Dynamic Software Product Lines

There are several approaches that propose the use of DSPL to provide reconfiguration behavior. However, they provide heavyweight solutions for general purpose platforms or applications, which are not applicable to resource-constrained pervasive devices, such as mobile devices or sensors [42,43]. In [42], the application of model-driven development and middleware technologies for supporting the development and operation of dynamic adaptive systems is presented. In [43], a feature-oriented approach to dynamically develop reconfigurable core assets is proposed. As in FamiWare [44], the authors of both proposals argue that dynamic systems can be considered as a product family line in which variabilities are bounded at runtime instead of at design time. Nevertheless, they do not provide a complete implementation of a reconfiguration process for WSN applications as FamiWare does.

More specifically, in pervasive systems, in [45], it is suggested that autonomic behavior can be achieved by using variability models at runtime. In that approach, feature models are used to guide the reconfiguration process in a similar way, as we do with CVL, but this solution is not proven for tiny devices, like sensors [44]. In [46], it is argued that DSPL can solve many challenges of the reconfiguration in intensive embedded systems, and the authors propose a runtime mechanism to change structural variability at execution time. They analyze the possible application of this proposal in four different application domains, but they do not propose a generic framework to provide context adaptation as FamiWare does. Finally, in [47], CANDEL (Context As dyNamic proDuct Line) is presented, a product line-based dynamic context management for pervasive applications. This is a generic context information representation framework that considers the context as a dynamic product line composed of context primitives specified as part of a metamodel. The development of this metamodel is presented as the first step towards a model-driven approach for the development of context-aware adaptive pervasive applications, but no model-driven process has been completed nor implemented.

Summarizing, all of these proposals promote, as does ours, the use of models to automatically adapt the systems to the context whilst maintaining a correspondence between models and code after the reconfiguration and ensuring that the reconfiguration is done safely. However, none of them provide an implementation that works for real sensor devices. Therefore, they cannot be used for security policy reconfiguration in WSNs, as we do with FamiWare.

## 8. Conclusions and Future Work

By combining the contributions of the FamiWare family of middleware and the contributions of the INTER-TRUST security framework, in this paper, we have presented an approach for building self-protected WSNs. Our approach allows us to: (1) build secure applications that run on heterogeneous nodes with limited capabilities; (2) dynamically negotiate the security level of the network nodes; (3) monitor the changes in the context in order to be able to react to those changes; (4) reconfigure the security level of the applications at runtime, and (5) perform the previous tasks using an efficient solution that is able to run in WSNs.

As regards future work, the first step will be, as we already mentioned in the Discussion section, the integration with the active and fuzz testing modules of INTER-TRUST to be able to verify at runtime that the system works as expected after the reconfiguration. These modules will be integrated in FamiWare as provided services in the same way as we have done in the work presented here, with the negotiation, security monitoring and security analysis modules.

Moreover, as FamiWare proposes an SPL approach to manage the large number of different devices in today's systems, as part of our future work, we plan to extend our approach to other Internet of Things devices, not only certain kinds of sensors, but also mobile devices. This extension for other kinds of devices will then be validated using other case studies, the e-voting and ambient assisted living applications.
